# Long-Term Outcomes of Obstetric Brachial Plexus Injury: A Single-Center Experience

**DOI:** 10.7759/cureus.73782

**Published:** 2024-11-15

**Authors:** Fatih Kurt, Tuba Erdem Sultanoglu

**Affiliations:** 1 Pediatrics, Duzce University, Duzce, TUR; 2 Physical Medicine and Rehabilitation, Duzce University, Duzce, TUR

**Keywords:** brachial plexus injury, clavicle fracture, macrosomia, oxytocin, panplexopathy

## Abstract

Obstetric brachial plexus injury (OBPI) is an important preventable complication of the birth process. While most cases recover in the early period, a substantial number result in sequelae. Despite established risk factors, there are cases that occur without any apparent risk factors. Our study aims to identify prenatal risk factors by examining OBPI cases born in our hospital and to provide insights into the prognosis of the disease by comparing cases with panplexopathy to those without.

We included 31 cases followed for OBPI in our hospital. Risk factors related to the infant and the mother were investigated. The range of motion (ROM) measurements of the affected upper extremity joints, muscle strength, atrophy, and scoliosis status were evaluated.

Of the cases, 71% were girls, and 61.3% had left-sided involvement. Additionally, 84.1% had risk factors, with oxytocin administration being the most frequently identified risk factor. In our study, we compared cases with panplexopathy to those without. Shoulder abduction, shoulder adduction, wrist flexion, and wrist extension restrictions were significantly more common in cases with panplexopathy. Furthermore, muscle atrophy was significantly more frequent in the panplexopathy group.

Since OBPI is a preventable injury, identifying risk factors is crucial. However, there are conflicting results regarding the most significant risk factor. Our study highlights that oxytocin administration is a very important risk factor for OBPI. We found that cases with panplexopathy had a worse prognosis, with more frequent ROM limitations and muscle atrophy.

## Introduction

The brachial plexus is a network of interlocked nerve components formed by the anterior branches of the cervical (C5-8) and thoracic (T1) sections of the spinal cord. This network innervates the muscles of the upper extremity and transmits cutaneous sensation [1]. Obstetric brachial plexus injury (OBPI) is damage to the brachial plexus occurring as a complication of the birth process. The incidence varies between 0.5-4 per 1,000 live births [2]. Known risk factors for OBPI include fetal macrosomia, gestational diabetes mellitus (GDM), instrument-assisted delivery, prolonged labor, fetal malpresentation, advanced maternal age, oxytocin use during labor, tachysystole, and excessive maternal weight gain during pregnancy. However, despite advances in healthcare, the fluctuating incidence of brachial plexus injury suggests the presence of other risk factors [3].

In OBPI, clinical presentations range from mild nerve damage to nerve root avulsion. OBPI results in three clinical syndromes: Erb's palsy due to injuries to C5-C6 and sometimes C7, Klumpke's palsy due to injuries to C8-T1, and total paralysis involving C5-C8 and T1 [4]. A significant portion of infants recover within the first six months with no or minimal functional deficits [3]. However, despite treatment strategies such as physiotherapy, splinting, microsurgical nerve reconstruction, interpositional nerve grafting, and distal nerve transfer, approximately 30% of patients suffer from severe sequelae [5].

OBPIs are traumas with a high rate of permanent sequelae, but their risk factors have not yet been fully identified. In this study, we aimed to determine prenatal risk factors by examining patients followed up for OBPIs in our hospital, and to gain insight into the prognosis of the disease by comparing cases with panplexopathy to those without panplexopathy.

## Materials and methods

The study included 31 patients who were born between 2008-2024 at Duzce University Practice and Research Hospital, diagnosed with OBPI based on clinical history, physical examination, and electromyography, and who were followed and treated. Patients diagnosed with cerebral palsy, progressive neurological disorders, metabolic diseases, or brachial plexus injury not related to birth trauma were excluded from the study. During the routine check-ups of the cases included in the study, physical examinations were performed by a pediatrician, height and weight measurements were taken, and percentiles were determined. Plasma 25-OH vitamin D, folate, and vitamin B12 levels were requested.

Range of motion (ROM) measurements of the affected upper extremity were evaluated using a universal goniometer while the patient was lying in the supine position. The range of motion was measured in degrees with a goniometer. Goniometric shoulder ROM measurements were performed in flexion, extension, abduction, adduction, internal rotation, and external rotation. The normal values for shoulder ROM were accepted as 0-180° for flexion, 0-60° for extension, 0-180° for abduction, 0-75° for adduction, 0-70° for internal rotation, and 0-90° for external rotation. The normal values for elbow ROM were accepted as 0-150° for flexion, 0-80° for wrist flexion, and 0-70° for wrist extension [6,7]. Muscle strength was measured using the Medical Research Council (MRC) Muscle Strength Assessment. The MRC Muscle Strength Assessment is a commonly used measurement method where muscle strength is graded on a scale of 0 to 5. If the muscle can fully resist maximum resistance after completing the movement against gravity, it is scored as 5 (normal); if the muscle can resist substantial but less than maximum resistance after completing the movement against gravity, it is scored as 4; if the muscle completes the normal range of motion against gravity but cannot resist any applied force, it is scored as 3; if the muscle completes the range of motion in a gravity-eliminated position, it is scored as 2; if there is a contraction without any movement in the joint, it is scored as 1; if there is no contraction in the muscle, it is scored as 0 [8]. Any atrophy in the affected extremity was recorded. The scoliosis assessment of the patients included in the study was performed using Adam's forward bend test. In this test, a prominent hump develops in the back or waist during forward bending due to spinal rotation if scoliosis is present. During Adam's forward bend test, the patient is positioned with both arms extended forward and the back parallel to the ground. In this position, asymmetric protrusion in the back/waist region and coronal imbalance were evaluated. Radiographic scoliosis images showing the entire spine in the anteroposterior direction were taken for patients suspected of having scoliosis to determine the degree of spinal curvature, and they were evaluated using the Cobb angle measurement method [9].

While performing sensory examinations in infant and toddler cases, responses were observed by stimulating different dermatomes with a cotton applicator or a broken tongue depressor. Sensory examinations of the preschooler cases, school-aged children and teens were performed through clinical evaluation and the Yes or No Test.

Descriptive statistics were presented as numbers and percentages. Demographic data were presented as Mean, Standard Deviation (SD), Median, and IQR. For normal distribution analysis, the bell curve was used when n>50, and the Shapiro-Wilk test was used when n<50. The relationship between two independent categorical variables was analyzed using Pearson's Chi-square test or Fisher’s Exact test. Bonferroni correction was applied for subgroup analyses. A significance level of P<0.016 was considered. The Mann-Whitney U test was used for the relationship between two independent numerical variables that did not show normal distribution, and the Student-t test was used for two independent numerical variables that showed normal distribution. The risk analysis between independent factors during and before birth and complete involvement in Electromyography (EMG) was presented with odds ratio and 95% Confidence Interval. Statistical analyses were performed using SPSS software for Windows, version 23 (IBM, Armonk, NY, USA). A significance level of P < 0.05 was considered.

## Results

A total of 31 cases with OBPI were included in the study. Between 2008 and 2024, a total of 12,268 births were performed at our center, and the incidence of OBPI was found to be 2.5 per 1,000 births. Of these, nine (29%) were male and 22 (71%) were female. The mean age of the cases was 9 ± 4.76 years (mean ± SD). One case (3.2%) had a height below the 3rd percentile, two cases (6.5%) were above the 97th percentile, and the others were between the 3rd and 97th percentiles. One case had a weight below the 3rd percentile, three cases were above the 97th percentile, and the others were between the 3rd and 97th percentiles. Twenty-five cases (80.6%) were born via normal spontaneous vaginal delivery (NSVD), four cases (12.9%) were delivered with the assistance of instruments (such as vacuum or forceps), and two cases were delivered via cesarean section (C/S). Five cases (16.1%) also had a concomitant clavicle fracture. The average age of the mothers during pregnancy was 29.95 ± 5.85 years (mean ± SD), and seven mothers (22.6%) were over 35 years old. During pregnancy, six mothers (19.5%) had gestational diabetes, two mothers had preeclampsia, and three mothers had hypothyroidism. One case was post-term (gestational age of 42 weeks or more), and 30 cases (96.8%) were term deliveries (37 weeks to 41 weeks and six days). Eleven cases (35.5%) were born macrosomic (>4000 g), while the others had normal birth weights. There weren’t any cases with fetal malpresentation, and three cases had a history of prolonged labor. Oxytocin was administered during delivery in 19 cases (61.3%). Five cases (16.1%) had no known risk factors. There weren’t any cases of Horner Syndrome in our cohort (Table 1). The mean plasma 25(OH)D levels of our cases were 13.11 ± 6.59 ng/ml (Mean ± SD), median plasma folate level was 6.70 (5-9.9) ng/ml (IQR 25-75), and the median plasma vitamin B12 level was 374 (236-461) pg/ml.

**Table 1:** Characteristics of the cases and their mothers during pregnancy, birth and postpartum period NSVD: normal spontaneous vaginal delivery, C/S: cesarean section, IQR: interquartile range

Parameters	n (%) = 31
(The number of Participants)	
Maternal Gestational Age (year)	
Mean ± SD	29.94 ± 5.85
Age (year)	
Mean ± SD	9 ± 4.76
Gender	
Male	9 (29)
Female	22 (71)
Height Percentile	
Low (<3)	1 (3.2)
Normal (3-97)	28 (90.3)
High (>97)	2 (6.5)
Weight Percentile	
Low (<3)	1 (3.2)
Normal (3-97)	27 (87.1)
High (>97)	3 (9.7)
NSVD (Normal Spontaneus Vaginal Delivery)	
Yes	25 (80.6)
No	6 (19.4)
C/S	
Yes	2 (6.5)
No	29 (93.5)
Instrumental Delivery	
Yes	4 (12.9)
No	27 (87.1)
Clavicula Fracture	
Yes	5 (16.1)
No	26 (83.9)
Gestational Diabetes Mellitus	
Yes	6 (19.4)
No	25 (80.6)
Birth Week	
Normal	30 (96.8)
Postterm	1 (3.2)
Birth Weight	
Normal	20 (64.5)
Macrosomic	11 (35.5)
Malpresentation	
Yes	0 (0)
No	31 (100)
Prolonged Labor	
Yes	3 (9.7)
No	28 (90.3)
Oxytocin administration	
Yes	19 (61.3)
No	12 (38.7)
Plasma 25(OH)D levels ( ng/ml)	
Mean ± SD	13.11 ± 6.59
Plasma Folate levels ( ng/ml)	
Median (IQR 25-75)	6.70 (5-9.9)
Plasma Vitamin B12 levels ( pg/ml)	
Median (IQR 25-75)	374 (236-461)

In 12 cases (38.7%) the right side was affected, and in 19 cases (61.3%) the left side was affected. Postnatal sensory loss was present in four cases (12.9%). Muscle atrophy was observed in five cases (16.1%), and scoliosis was present in nine cases (29%). One case did not receive any treatment, seven cases (22.6%) underwent surgery and received physical therapy, and the others received only physical therapy. Twenty cases (64.1%) were still undergoing physical therapy. The median age at the start of treatment was median five months (IQR 2-12 months). In the EMG evaluations of the cases, it was observed that six cases (19.4%) had no upper trunk injury. Seventeen cases (54.8%) had mild, three cases had moderate, and five cases had severe upper trunk injury. In 20 cases (64.5%), there was no middle trunk injury, while four cases had mild, three cases had moderate, and four cases had severe middle trunk injury. In 15 cases (48.4%), there was no lower trunk injury, while 10 cases (32.3%) had mild, two cases had moderate, and four cases had severe lower trunk injury. Panplexopathy was detected in 10 cases (32.4%) (Table 2).

**Table 2:** Clinical characteristics, electromyography findings and treatment modalities of the cases IQR: interquartile range

Parameters	n (%)=31
(Postnatal Control)	
Loss Of Sensation	
Yes	4(12.9)
No	27 (87.1)
Atrophy	
Yes	5 (16.1)
No	26 (83.9)
Scoliosis	
Yes	9 (29)
No	22 (71)
Electromyography upper truck injury	
None	6 (19.4)
Mild	17 (54.8)
Moderate	3 (9.7)
Severe	5 (16.1)
Electromyography middle truck injury	
None	20 (64.5)
Mild	4 (12.9)
Moderate	3 (9.7)
Severe	4 (12.9)
Electromyography lower truck injury	
None	15 (48.4)
Mild	10 (32.3)
Moderate	2 (6.5)
Severe	4 (12.9)
Electromyography Panplexopathy	
Yes	10 (32.3)
No	21 (67.7)
Operation	
Yes	7 (22.6)
No	24 (77.4)
Physical Therapy	
Yes	30 (96.8)
No	1 (3.2)
Treatment Start Age (Months)	
Median (IQR 25-75)	5 (2-12)
Physical Therapy Continuation	
Yes	20 (64.5)
No	11 (35.5)

In another important aspect of our study, when comparing cases with panplexopathy to those without panplexopathy, no significant differences were found in terms of gender, birth weight, mode of delivery, prolonged labor, administration of oxytocin, or the presence of clavicle fracture (Table 3).

**Table 3:** Comparison of independent variables before and during birth in cases with panplexopathy and cases without panplexopathy *p value was obtained from the Fisher's Exact test. ** p value was obtained from the Student's t-test. *** p value was obtained from the Mann-Whitney U test. **** p value was obtained from the Chi-Square test. C/S: cesarean section, IQR: interquartile range

Parameters	Panplexopathy		Total	Value	OR	95% CI
	n (%)		n=31			Lower-Upper
	No	Yes				
Gender					4.250	0.816-22.133
Male	4 (44.4)	5 (55.6)	9	0.105*		
Female	17 (77.3)	5 (22.7)	22			
Maternal Gestational Age					-	-
(Mean ± SD)	29.67 ± 6.10	30.50 ± 5.56		0.718**		
Age (year)						
Median (IQR 25-75)	7 (4.50-13.50)	10 (7-13.50)		0.396***		
Height Percentile					-	-
<3 p	1 (100)	0 (0)	1	0.453***		
Normal	18 (64.3)	10 (35.7)	28			
>97 p	2 (100)	0 (0)	2			
Weight Percentile					-	-
<3 p	0 (0)	1 (100)	1	0.335***		
Normal	19 (70.4)	8 (9.6)	27			
>97 p	2 (66.7)	1 (33.3)	3			
Spontaneous Vaginal Delivery					6.333	0.920-43.620
No	2 (33.3)	4 (66.7)	6	0.067*		
Yes	19 (76)	6 (24)	25			
C/S					0.450	0.025-8.027
No	20 (69)	9 (32)	29	0.548*		
Yes	1 (50)	1 (50)	2			
Instrumental Delivery					8.571	0.761-96.525
No	20 (74.1)	7 (25.9)	27	0.087*		
Yes	1 (25)	3 (75)	4			
Gestational Diabetes Mellitus					2.571	0.415-15.918
No	18 (72)	7 (28)	25	0.358*		
Yes	3 (50)	3 (50)	6			
Preeclampsy					2.222	0.125-39.640
No	20 (69)	9 (31)	29	0.548*		
Yes	1 (50)	1 (50)	2			
Birth Week					0.651	0.024-17.399
Normal	20 (66.7)	10 (33.3)	30	0.677*		
Postmaturity	1 (100)	0 (0)	1			
Birth Weight					1.333	0.281-6.325
Normal	14 (70)	6 (30)	20	0.510*		
Macrosomic	7 (63.6)	4 (36.4)	11			
Prolonged Labor					1.056	0.084-13.226
No	19 (67.9)	9 (32.1)	28	0.704*		
Yes	2 (66.7)	1 (33.3)	3			
Oxytocin administration					1.750	0.352-8.712
No	9 (75)	3 (25)	12	0.697*		
Yes	12 (63.2)	7 (36.8)	19			
Clavicula Fracture					1.500	0.208-10.794
No	18 (69.2)	8 (30.8)	26	0.528*		
Yes	3 (60)	2 (40)	5			

There were also no significant differences in plasma 25(OH)D, vitamin B12, folate levels, age at the start of treatment, sensory loss, or the development of scoliosis (Table 4). However, cases with panplexopathy had significantly more frequent restrictions in shoulder abduction, shoulder adduction, hand flexion, and hand extension. Muscle atrophy was significantly more frequent in the panplexopathy group compared to the other group (Tables 4, 5).

**Table 4:** Comparison of range of motion (ROM) in cases with panplexopathy and cases without panplexopathy *p value was obtained from the Fisher's Exact test

Parameters	Panplexopathy		Total	p value
	n (%)		n=31	
	No	Yes		
Shoulder Abduction				
Normal	17 (85)	3 (15)	20	0.013*
Limited	4 (36.4)	7 (63.6)	11	
Shoulder Adduction				
Normal	17 (81)	4 (19)	21	0.040*
Limited	4 (40)	6 (60)	10	
Shoulder Flexion				
Normal	18 (78.3)	5 (21.7)	23	0.074*
Limited	3 (37.5)	5 (62.5)	8	
Shoulder Extension				
Normal	17 (77.3)	5 (22.7)	22	0.105*
Limited	4 (44.4)	5 (55.6)	9	
Shoulder External Rotation				
Normal	16 (76.2)	5 (23.8)	21	0.222*
Limited	5 (50)	5 (50)	10	
Shoulder Internal Rotation				
Normal	15 (75)	5 (25)	20	0.423*
Limited	6 (54.5)	5 (45.5)	11	
Elbow Flexion				
Normal	15 (75)	5 (25)	20	0.423*
Limited	6 (54.5)	5 (45.5)	11	
Elbow Extension				
Normal	13 (76.5)	4 (23.5)	17	0.441*
Limited	8 (57.1)	6 (42.9)	14	
Wrist Flexion				
Normal	20 (76.9)	6 (23.1)	26	0.027*
Limited	1 (20)	4 (80)	5	
Wrist Extension				
Normal	20 (76.9)	6 (23.1)	26	0.027*
Limited	1 (20)	4 (80)	5	

**Table 5:** Comparison of clinical characteristics in cases with panplexopathy and cases without panplexopathy *p-value obtained from the Fisher's Exact test.

Parameters	Panplexopathy		Total	p value
	n (%)		n=31	
	No	Yes		
Erb Paralysis				0.002*
No	6 (40)	9 (60)	15	
Yes	15 (93.8)	1 (6.3)	16	
Klumpke Paralysis				0.704*
No	19 (67.9)	9 (32.1)	28	
Yes	2 (66.7)	1 (33.3)	3	
Total Paralysis				0.013*
No	17 (85)	3 (15)	20	
Yes	4 (36.4)	7 (63.6)	11	
Loss of Sensation				
No	20 (74.1)	7 (25.9)	27	0.087*
Yes	1 (25)	3 (75)	4	
Atrophy				
No	20 (76.9)	6 (23.1)	26	0.027*
Yes	1 (20)	4 (80)	5	
Scoliosis				
No	17 (77.3)	5 (22.7)	22	0.105*
Yes	4 (44.4)	5 (55.6)	9	

## Discussion

OBPI cases born at Duzce University Practice and Research Hospital were examined in this study. These cases are injuries with a high probability of sequelae. When analyzing the risk factors for OBPI in our cohort, the most frequently identified risk factor was the administration of oxytocin. This was followed by macrosomic birth, maternal GDM, and instrument-assisted delivery. When panplexopathy cases were compared to other cases, limitations in shoulder abduction and adduction, wrist flexion and extension angles were detected significantly more frequently. Additionally, muscle atrophy was significantly higher in panplexopathy cases.

Lalka et al. reported that 55% of their cases did not have any risk factors [10]. Hardie et al. reported in their study that 82% of OBPI cases had a risk factor [11]. In our study, 84.1% of the cases had risk factors. The presence of risk factors may vary depending on the characteristics of the cohort studied. Studies have reported that approximately 66-75% of the cases recovered completely [12]. In our study, complete recovery was found in 11 (35%) of the cases, and there was limitation in muscle strength and joint range of motion in the other cases. This situation is due to the continued treatment of 20 (64%) cases. In the Helsinki protocol, it is recommended to initiate passive exercises immediately for cases of OBPI. Parents' belief that their children will recover spontaneously may lead to a delay in treatment [13]. Unfortunately, the median age of starting treatment in our cases is 4.5 months. The delay in treatment also contributes to the low rate of full recovery. Bar et al. reported that 27% of the cases with OBPI had a macrosomic birth history (over 4000 g), while 4.8% in the control group had a macrosomic birth history and macrosomia was a very important risk factor [14]. In our study, 35.5% of the cases had a history of macrosomic birth.

Grahn reported that 28% of mothers of cases with OBPI had GDM [15]. In our study, GDM was detected in 19.4% of the cases. This discrepancy may be due to pregnant women who do not regularly attend their check-ups or refuse the glucose tolerance test.

The literature indicates that OBPI cases are more frequent in girls and that the right side is more commonly affected [16]. However, in our study, 71% of the cases were female, and 61.3% had left-sided involvement.

Linder et al. reported that C/S is a protective factor against OBPI [17]. Johnson et al. found that 9% of cases with OBPI occurred via C/S [18]. In our study, only 6.5% of cases were delivered by C/S. Studies have indicated that advanced maternal age (>35 years) is a risk factor for OBPI [19]. In our study, 22.6% of the mothers were over 35 years old at the time of delivery.

Louden et al. reported that oxytocin was used during delivery in 79% of cases with OBPI and that oxytocin use increased the likelihood of OBPI by 2.5 times [3]. Yenigül et al. reported that oxytocin was administered during birth in 66.7% of OBPI cases. Oxytocin administration was an independent risk factor for clavicle fracture concurrent with OBPI [20]. Lau et al. reported that oxytocin administration is a significant risk factor for shoulder dystocia, which can lead to OBPI [21]. Tandon et al. reported in their study on primiparous mothers that OBPI was more frequently observed in babies of mothers who received oxytocin [22]. In our study, oxytocin was used during delivery in 61.3% of cases. The most frequently identified risk factor in our cohort was oxytocin use, which increased the likelihood of panplexopathy by 1.75 times.

Lalka et al. identified shoulder dystocia and instrument-assisted delivery as the most significant risk factors for OBPI, reporting that 4.8% of cases involved instrument-assisted delivery. They found that the likelihood of OBPI increased 12.2 times with instrument-assisted delivery [10]. In our study, 12.9% of cases had a history of instrument-assisted delivery, which increased the likelihood of panplexopathy by 8.5 times. This discrepancy may be due to differences in national conditions and the experience of the personnel using the instruments.

Gandhi et al. reported that clavicular fractures accompanied 7.8% of OBPI cases. They noted that the incidence of OBPI was similar in cases with shoulder dystocia and clavicular fractures compared to those without clavicular fractures. However, shoulder dystocia without clavicular fracture was found to be an independent risk factor for OBPI [23]. In our study, clavicular fractures accompanied 16.1% of OBPI cases. This variation may be related to differences in the experience of the delivery staff and the use of effective assistive techniques. When examining panplexopathy, no significant difference was found between the panplexopathy group and the non-panplexopathy group regarding clavicular fractures. However, the presence of a clavicular fracture was found to increase the risk of panplexopathy by 1.5 times.

Although there are many studies related to OBPI, few compare cases with panplexopathy to those without panplexopathy. Studies have reported that panplexopathy cases have a worse prognosis and less spontaneous recovery [24]. Caron et al. classified cases according to the Narakas classification, noting that Class III cases did not show improvement in shoulder abduction, elbow flexion, or elbow extension, while Class I cases showed more improvement [25]. In our study, shoulder abduction limitation was detected significantly more frequently in cases with panplexopathy than in the group without panplexopathy, but no significant difference was detected in terms of elbow flexion and extension limitation.

Çelik et al. reported significantly lower upper extremity developmental skill capacity and gross motor function quality in patients with panplexopathy compared to those without [26]. In our study, muscle atrophy was found to be significantly higher in patients with panplexopathy than in the other group. Loss of function in dexterity and gross motor movements occurs, probably due to muscle atrophy in the upper extremity. One of the main goals in OBPI treatment is to ensure hand movements and prevent the development of chronic contractions. In our study, it was found that the patients with panplexopathy had significantly more limitations in hand flexion and extension movements. Although no significant difference was detected, scoliosis was seen to be more common in the panplexopathy group. No significant difference was found between the two groups in terms of joint range of motion limitation in shoulder flexion, shoulder extension, shoulder internal rotation, shoulder external rotation, elbow flexion and elbow extension.

In our study, 71% of the participants were female. Despite the lack of significant differences between genders, being male was found to increase the likelihood of panplexopathy by 8.5 times. Additionally, maternal GDM increased the likelihood of panplexopathy by 2.5 times, preeclampsia by 2.2 times, and NSVD by 6.3 times. The median time to start treatment for cases with panplexopathy was 4.5 months, compared to six months for cases without panplexopathy. Although the time to start treatment was shorter for panplexopathy cases, the difference was not statistically significant.

There were some limitations in our study. First, the number of patients analyzed was limited. Second, this study was a single-center retrospective study. Future studies with larger sample sizes are needed to validate the results of this study. Additionally, long-term follow-up of patients, along with prospective assessments using disability scales after plexus injury, would be more beneficial for generalizing the study's findings.

## Conclusions

Our study is significant because it highlights the association between oxytocin administration and OBPI. We found that cases with panplexopathy have a worse prognosis, with significantly more frequent restrictions in joint range of motion and significantly more widespread muscle atrophy. We recommend evaluating OBPI cases in terms of scoliosis and muscle atrophy during follow-up.
